# Implementing large-scale workforce change: learning from 55 pilot sites of allied health workforce redesign in Queensland, Australia

**DOI:** 10.1186/1478-4491-11-66

**Published:** 2013-12-11

**Authors:** Susan A Nancarrow, Alison Roots, Sandra Grace, Anna M Moran, Kerry Vanniekerk-Lyons

**Affiliations:** 1 School of Health and Human Sciences, Southern Cross University, Military Road, East Lismore, NSW 2480, Australia

**Keywords:** Role redesign, Workforce change, Implementation, Logic model, Proposition testing, Mixed methods, Workforce flexibility, Allied health, Inductive logic reasoning

## Abstract

**Background:**

Increasingly, health workforces are undergoing high-level ‘re-engineering’ to help them better meet the needs of the population, workforce and service delivery. Queensland Health implemented a large scale 5-year workforce redesign program across more than 13 health-care disciplines. This study synthesized the findings from this program to identify and codify mechanisms associated with successful workforce redesign to help inform other large workforce projects.

**Methods:**

This study used Inductive Logic Reasoning (ILR), a process that uses logic models as the primary functional tool to develop theories of change, which are subsequently validated through proposition testing. Initial theories of change were developed from a systematic review of the literature and synthesized using a logic model. These theories of change were then developed into propositions and subsequently tested empirically against documentary, interview, and survey data from 55 projects in the workforce redesign program.

**Results:**

Three overarching principles were identified that optimized successful workforce redesign: (1) drivers for change need to be close to practice; (2) contexts need to be supportive both at the local levels and legislatively; and (3) mechanisms should include appropriate engagement, resources to facilitate change management, governance, and support structures. Attendance to these factors was uniformly associated with success of individual projects.

**Conclusions:**

ILR is a transparent and reproducible method for developing and testing theories of workforce change. Despite the heterogeneity of projects, professions, and approaches used, a consistent set of overarching principles underpinned success of workforce change interventions. These concepts have been operationalized into a workforce change checklist.

## Background

The Australian healthcare system faces a number of challenges that test its ability to deliver effective, efficient, and responsive services to the population. These challenges are well documented and include: an increasing demand for services [1,2], a growing prevalence of chronic disease [3,4], escalating service costs [5], diminishing workforce availability [6-8], and changing community expectations [7,9].

In response to these challenges, the report *The Australian Allied Health Workforce* - *An Overview of Workforce Planning Issues*[10] recommended investigation into ways that the allied health workforce could be reformed to promote sustainable allied health services and viable models for service delivery. This reform agenda was supported by Queensland Health (QH) through their Health Practitioners Models of Care (QH MoC) strategy, which implemented a number of innovative workforce redesign and reform projects to trial new models of care (MoC) delivery.

‘Models of care’ is a broad concept that describes the best way to deliver patient care services to a specific population [11]. The objective of MoC is to ‘ensure people get the right care, at the right time, by the right team and in the right place’ [11]. The MoC approach to service delivery is designed to align the healthcare workforce with services that are consumer focused, aligned with service delivery plans, multidisciplinary, and holistic in their approach to care. This MoC approach has been taken up nationally through the Australian Health Ministers’ Advisory Council and three national workforce planning committees - the Australian Medical Workforce Advisory Committee, the Australian Health Workforce Advisory Committee (nursing and allied health), and the Australian Health Workforce Officials’ Committee [12], and is implemented by individual jurisdictions to meet their service and workforce planning requirements.

The QH MoC strategy supported the introduction of new MoC that:

•used allied health scopes of professional practice to their fullest extent

•advanced or extended scopes of practice

•better utilized support staff (assistant staff)

•partnered with internal and/or external staff

•used multidisciplinary approaches and integrated health services across the health care delivery continuum.

This strategy facilitated workforce reform through the development of an organizational environment that encouraged, enabled, and sustained these new MoC. Initiatives were undertaken to enhance leadership capacity, workplace culture, training and education, and interprofessional collaboration, as well as to resolve human resource, industrial, and legislative issues. One arm of this strategy involved sponsoring demonstration projects that more efficiently used the skills of allied health professionals (AHPs) and assistants through alternative models of service delivery. These demonstration projects sought to develop, trial, and embed new MoC that delivered best practice, improved patient outcomes, enhanced workforce sustainability, and managed demand within allocated resources. In particular, they examined and evaluated: 1) the best use of full /advanced and extended scope of practice roles for AHPs and use of allied health assistants (AHAs); 2) the potential for new and different roles; 3) the sharing of competencies and tasks to decrease duplication; 4) the use of technology to enhance service delivery; and 5) the increased coordination of services. One such project involved the assessment of patients on an orthopedic waiting list to see whether non-urgent issues could be managed conservatively by a podiatrist [13].

Queensland Health funded two rounds of MoC demonstration projects between 2009 and 2013 with the first 30 projects completed in 2011. The second round commenced in 2011 and involved 34 new projects including five continuing from Round 1. Each model of service delivery was trialed for up to two years. To our knowledge, this is the largest single workforce redesign project in Australia.

There is a dearth of theory relating specifically to workforce change. A recent review of workforce change instruments undertaken as part of a large, workforce change project [14] found that few of these instruments were evidence-informed, nor had they undergone any formal evaluation. There have been a number of recent, large-scale, role-redesign projects including the National Health Service (United Kingdom) Changing Workforce Program, which incorporated the Accelerated Development Program [15-17]; however, neither of these programs captured systematic learning or codified processes and mechanisms for workforce change in a reproducible way. Health Workforce Australia recently published an evaluation of a large-scale change program of workforce innovation in caring for older people [18]. This evaluation went some distance in creating a model for workforce change by providing a conceptual framework, demonstrating evidence of innovation, discussing change management, stakeholder engagement, and program design. One Australian study used program logic in workforce research to develop a logic model for sustainable workforce retention in rural and remote health [19]. The study highlighted the impact of culture on the ability of individuals and organizations to change; the importance of strong clinical leadership in implementing organizational change and creating a culture that is responsive to change.

The aim of this study was to systematically explore the learning from the QH MoC projects to develop guidelines to inform future workforce change projects. One of the unique features of this project was that it drew on a range of different types of role redesign, professional backgrounds, and contexts. Much of the existing literature on workforce redesign is based on a single setting, profession, or type of role.

## Methods

The learning of interest from this study was an understanding and codification of the reproducible processes, or mechanisms, that lead to successful workforce reform. This required a methodology that could link the way the intervention was delivered to the specific outputs or outcomes from that intervention, or in other words, the theories to explain change. At the same time we needed to synthesize data from a large number of heterogeneous projects and literature and then manage and process that information in a transparent way. A number of methodological approaches facilitate this type of descriptive, causal analysis. Donabedian’s structure - process - outcome triad is designed to look beyond the outcome and examine the modifiable ingredients that lead to the achievement of that outcome [20]; however, it is not an evaluation tool *per se*. Logic models are a well-developed tool for graphically illustrating the various project components to identify the drivers, contexts, mechanisms or activities, outputs and outcomes [21]. However, logic models do not provide a transparent way to link together the component activities and outcomes to produce theories.

Two contemporary, theory-based evaluation approaches are Realistic Evaluation and Theories of Change. Realistic Evaluation explores the relationship between contexts, mechanisms, and outcomes within a structured theoretical framework [22] to describe what intervention works for whom, and in what circumstances. Theories of Change is an increasingly popular tool in program evaluation to explore the relationships between activities and outcomes and to look at how and why the changes occur [23]. At face value, there are several similarities between these two methods; however, Blamey and Mackenzie juxtapose the approaches, highlighting the differences between them and the challenges of each [24].

Neither of these approaches was able to be applied fully to this study for several reasons. The MoC projects had already commenced, and many had finished; thus, the theories needed to be developed retrospectively. Within the MoC evaluation, there were several different types of projects and a range of different drivers. The complexity of project types meant that we perceived Realistic Evaluation to be too ‘fine-tuned’ to cope with the macro and meso level data and concepts. Theories of Change appeared to be better equipped to deal with this complexity, but it needed an underlying theory with which to begin. Furthermore, neither method was well described or used consistently in the published literature in a way that was easy to adopt for this study [24].

**Figure 1:**
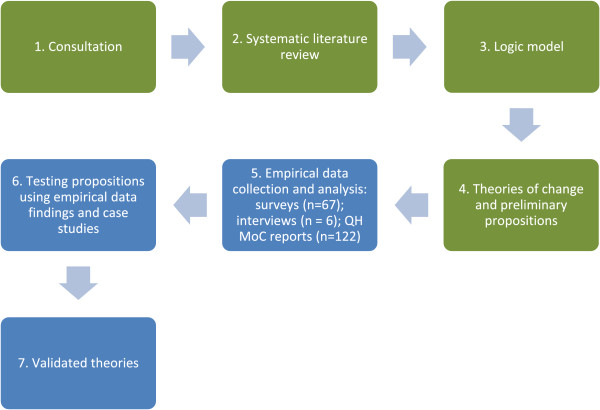
Steps involved in Inductive Logic Reasoning.

Consequently, we developed Inductive Logic Reasoning (ILR) to address these limitations. The principle analytical tool was the logic model that was used to systematically extract and organize the data under the headings drivers, contexts, mechanisms (barriers and facilitators), outputs, and outcomes [21]. The iterative development and modification of these logic models provided a structured, systematic, and transparent way to document the findings from the multiple data sources. The approach is summarized in Figure 1.

The research was undertaken in two phases (Figure 1): Phase one included steps 1 to 4 and Phase two included steps 5 to 7. The analytic framework was developed following detailed consultation with steering committee members and those involved in the MoC projects.

Phase one involved a systematic review of the MoC literature which was extracted into an initial logic model describing the drivers, contexts, mechanisms, and outcomes underpinning workforce change. The systematic review aimed to identify research relevant to the MoC projects. This began with a search of systematic reviews of new models of care that were supplemented with specific, individual, high quality studies of interventions specifically identified within the different types of MoC. The search strategy used is [25] presented in Table 1. The Cochrane Collaboration’s Effective Practice and Organisation of Care (EPOC) reviewing guidelines [26] were used to develop a data extraction framework because they are specific to health service interventions. All members of the research team participated in the data extraction using a ‘fillable’ form in a Google Documents shared spread sheet.

**Table 1:** Sampling framework for the systematic review of models of care (MoC) literature using STARLITE

	
S: Sampling Strategy	Selective: systematic reviews and intervention studies specific to the Queensland Health (QH) MoC projects taxonomy
T: Type of literature	Any kind of literature, qualitative and quantitative studies, and gray literature
A: Approaches	Subject searching, citation searching, internet searching, gray literature, available documents from QH
R: Range of Years (start date: end date)	2000 to 2012
L: Limits	English, human
I: Inclusions and exclusions	Inclusion: allied health, nursing, leadership, models of care, integrative health care, Exclusions: developing country health care, educational projects, school-based services, not related to new models of care delivery, clinical training, interprofessional education, recruitment, retention
T: Terms used	Allied health, models of care, new roles, service redesign, role redesign, practice models, integrated delivery, skill development, collaboration, role substitution, interprofessional working, extended scope, health care assistant, screening services, triage
E: Electronic sources	CINAHL(EBSCO), MEDLINE (EBSCO), Health Source: Nursing/Academic (EBSCO), AMED (EBSCO), PsycINFO (EBSCO), ERIC

Four types of literature were accessed: systematic reviews; qualitative reviews of specific interventions; quasi-experimental design studies; and diagnostic test studies relevant to the ‘triage, assessment and treatment’ models of care projects.

All studies were assessed for quality using criteria from the Critical Appraisal Skills Programme (CASP) [27] for systematic reviews, qualitative research, and diagnostic test studies, and the Joanna Briggs Institute Meta-Analysis of Statistics Assessment and Review Instrument, Randomized Trials and Pseudo-randomized Trials [28]. Findings from identified studies were extracted to a series of tables (drivers, barriers, facilitators, outcomes, outputs). Themes were identified using a constant comparative method [29] and, once identified, were coded in each study. A thematic synthesis was used to look for common patterns across studies [30], and the findings were first summarized into the logic model for each type of workforce redesign and then were summarized as an over-arching synthesis for all workforce redesign.

The definitions of the logic model components are described in Table 2. Few published approaches to logic models incorporate the drivers; however, we found that the drivers were closely related to the outcomes and helped develop a coherent pathway linking the components of the logic model together. The logic model is organized to show the relationships between the component parts (Table 3). For instance, working across the second row of the logic model illustrates how the ‘workforce drivers’ required a context of existing workforce support and mechanisms that promoted engagement to produce better ‘workforce outcomes’. The bottom row of the logic model (‘remove strategic uncertainty’ and ‘project management’) was not linked directly to drivers or outcomes, but provided the project management context that is essential for the success of any workforce change project.

**Table 2:** Definitions used to develop the logic models

**Concept**	**Definition**
Drivers	The underlying motivation for the changes under review, and tend to answer the question ‘why is this intervention taking place?’ The drivers and outcomes are important, and tend to form the ‘anchors’ for the logic model. Drivers and outcomes should be closely related.
Contexts	The physical, material, organization and/or social environment in which the change is taking place. These become the enabling/disabling environments for the change to take place.
Mechanisms	Mechanisms are a complex idea to distill on their own. Instead, we extracted the barriers and facilitators to change. Often (but not always) the barriers and facilitators are the opposite of each other, and when written as a positive statement, they become the mechanisms to support change.
Outputs	The outputs as the material or measurable products of undertaking the process or project under investigation. They tend to be tangible, countable, and relatively uncontentious products of the research and they are often the clearly codifiable components of the process.
Outcomes	Outcomes are the changes resulting from the intervention, and should be closely related to the drivers. Outcomes often require a formal process of evaluation/research to capture in a meaningful way.

**Table 3:** The logic model and developing theories of change

**Drivers**	**Contexts**	**Mechanisms**	**Outputs**	**Outcomes**
**Workforce drivers**	**Support from existing workforce**	**Engagement**		**Workforce outcomes**
Workforce recruitment and retention, skills shortages, workforce participation rates (particularly in rural areas), inefficient use of staff, improved models of care, improved quality	Support from ‘powerful elites’, inter-disciplinary support, inter-institutional support, willingness to delegate, organizational culture that is supportive of change.	Team buy in, corporate sponsorship and senior management support, medical support, engagement of staff/clinicians		Working to full scope of practice, wider uptake of role, enhanced team processes/working, engagement of rural practitioners, improved relationships
**Policy drivers**	Clarity of role definition, supportive human resource (HR) policies, appropriate legislative scaffolding, funding secured, clear strategic direction, governance structures established	**Resources to facilitate new roles**	New resources to support the development and implementation of new roles, Codified processes, Training, Creation of new positions.	**Service outcomes**
Industrial agreements, Productivity Commission health workforce position paper, need to meet national targets		Sufficient funding, data quality and compatibility, dedicated resources and facilities, sufficient staffing, sufficient time, appropriate tools to support implementation, access to training and support, Calderdale Framework, evidence of success from other areas (literature/other sites) and resources.		Implementation of new roles and ability to work to full scope of practice, acceptance of new roles, better understanding of roles, improved service efficiencies increased service capacity, reduced waiting lists, cost savings,
**Population drivers**		**Engagement**		**Population/patient outcomes**
Demographics of the population, changing health needs, changing patient expectations, need to increase patient safety, need to increase accessibility.		Local engagement, patient engagement.		Improved patient satisfaction & functional outcomes, improved pathways of care, improved diagnostic accuracy, Improved accessibility.
**Service drivers**				
Waiting lists, address service gaps, improved patient outcomes, improved efficiency of services, meeting demand/overcoming shortages				
	**Remove strategic uncertainty**	**Project management**		Sustainability and transfer of learning, other service redesign spin-offs, organizational learning, understanding the change process.
	Minimize disruption from organizational changes, limit competing projects/priorities, limit implementation of new roles in times of substantial changes.	Clearly defined problem and scope, realistic project expectations, consistent expectations, skilled project management, project manager on site and connected to management and reporting structures.		

From the logic model, we developed a series of testable, proposition statements, or preliminary theories of change. The proposition statements were developed by linking the outcomes to the drivers, contexts and/or mechanisms (Table 4). Five broad propositions were developed, each relating to a specific outcome category. Within these, specific sub-statements were developed that were tested in phase two.

**Table 4:** Proposition testing

***Proposition***	***Proposition supported *** **( *Y */ *N *)**
*1. Better sustainability of the model of care* (*MoC*) *is associated with*:	
1.1. Full engagement of all key stakeholders first	Yes
1.2. Bottom-up drivers (rather than top-down)	Yes
1.3. Top-down support - Legislative support to drive, underpin, and sustain the new MoC created	Yes
1.4. Legislative scaffolding to reinforce the new MoC, such as award and pay structures, that are supported in industrial agreements and ratified at the highest possible levels of government to avoid undermining by professional boundary arguments	Yes
1.5. Codification of the processes, practices and training used to implement the role	Yes
1.6. Having powerful allies to drive the role forward	Yes
1.7. Implementing new MoC that are appropriate for the context (including local, geographic, population, clinical, professional, regulatory contexts)	Yes
*2. More*/*less efficient use of the role is associated with*:	
2.1. Clearly defined roles within the MoC	Yes
2.2. Clearly defined and understood, unambiguous delegatory or allocatory MoC	Yes
2.3. Delegating practitioners having confidence in delegation, which comes from understanding the roles, training, and competencies of the practitioners to whom they are delegating	Yes
2.4. Trust, derived from time and exposure to the new model of care, is important for establishing appropriate delegation/collaboration/referring practices	Yes
3. *Greater staff satisfaction is associated with*:	
3.1 Better career development opportunities	Yes
3.2 Role clarity	No
3.3 Appreciating value in/impact of the role	Yes
3.4 Appropriate support for the development and implementation of the MoC	Yes
*4. Better patient outcomes are associated with*:	
4.1. Greater engagement of patients in the decision making associated with their care delivery	Yes
4.2. Putting the patient at the centre of the MoC, rather than the practitioner	Yes
4.3. Providing any care or service where the alternative is no service, or a long waiting list	Yes

Phase two involved testing the propositions against the empirical data arising from the QH MoC projects. Detailed, ongoing consultation took place with both QH and the project steering committee, selected for their expertise in workforce change, regarding the development of these initial theories. The final output was an empirically tested set of propositions and principles to inform workforce change, and a *Workforce Change Checklist* to guide workforce change projects.

Three sources of project data were used for Phase two of the research: documents developed by the individual MoC projects, an E-survey, and key stakeholder interviews.

An E- survey was developed and circulated using Qualtrics survey software to all of the project sponsors and project officers involved with each of the MoC projects. The survey included around ten open-ended questions (there were slight differences between the sponsor and project officer surveys). The questions were based on the logic model structure in Table 3 with additional questions about project sustainability and transfer. Three reminders were circulated at weekly intervals from the initial email.

Interview participants were purposively sampled to ensure representation of project types and population densities (urban, rural). Semi-structured interviews were used to obtain greater depth of understanding of the processes of implementation of the projects, capacity building, sustainability, and identify any novel approaches. Interviews were recorded, summarized, and coded using the constant comparative method to identify themes and trends [29,30].

All projects produced a substantial amount of documentary data including concept briefs, project plans, feasibility studies, monthly reports, quarterly reports, project completion reports, and published literature. Project completion reports were the principal source of data; for incomplete projects the most recent quarterly report and any other reports deemed relevant were used. A data extraction framework was created to evaluate the data from each project for its degree of support for, or divergence from, the propositions as well as to identify project drivers, facilitators, barriers, outputs, and outcomes.

Team members extracted data directly from the source documents into a pre-coded template that comprised specific questions relating to the headings identified above. To do this, we developed a customized, fillable form within Google Documents [see Additional file 1]. In this sense, the method can most closely be related to template analysis [31], in which an *a priori* template is used to code the data. In our case, we cut and pasted the documentary data directly into the Google form against the *a priori* questions (themes). The Google form collated the data in a spread sheet. We extracted the raw data directly from the Google spread sheet and thematically analyzed it to form a new logic model and to test each of the propositions. The propositions were tested qualitatively by looking at the volume and strength of supporting or refuting data against each statement. Propositions with insufficient data from which to draw conclusions were not supported. Additional file 2 illustrates an example of the data extracted for a single project (project 14).

Ethics approvals were obtained from the Human Research Ethics Committees of Southern Cross University and Queensland Health.

## Results

### Systematic review

**Figure 2:**
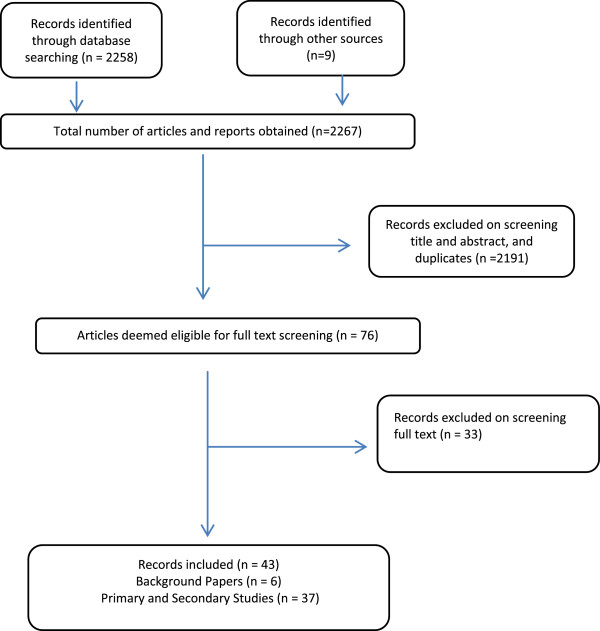
**PRISMA Summary of paper screening process**[32]**.**

This search strategy produced 2,267 articles and reports. The titles and abstracts of the identified literature were screened for relevance. Following removal of duplicates and initial screening 76 articles remained. These articles were subjected to full text screening following which 43 articles remained; 37 of these met the criteria for inclusion in the data extraction process. Six were not primary or secondary studies and, therefore, were used only for background purposes. Figure 2 displays the numerical summary of the papers resulting from each stage of the screening process.

### Response rates

The MoC projects involved an extensive range of health professions. AHPs were the primary disciplines involved; however, in many cases the implementation of the changes also required involvement from medicine and nursing. The primary disciplines involved were physiotherapy (PT), occupational therapy (OT), social work (SW), dietetics, podiatry, pharmacy, psychology, speech therapy, audiology, clinical measurement, medical imaging, orthoptics, and oral health. The total number of staff involved in these projects was estimated at more than 500. A full list of the MoC projects is included as Additional file 3.

One hundred twenty-two (122) documents were included in the data extraction process. Of 103 surveys distributed, 84 responses were received, of which 67 were usable. Responses were received from project sponsors and officers, and others including clinicians and clinical leaders working within the projects, steering committee members, trainers, managers, and department directors associated with the projects. Fifteen projects were identified for further follow up by interview, however in eight cases there was no one available to provide additional information about the projects due to staff turnover. Participants from six of the remaining seven projects agreed to participate in interviews.

Additional file 4 presents a summary of the outcomes of all of the projects based on success, sustainability, and replication of the project. Additional file 5 shows the results of the data extracted against each of the propositions, illustrating the confirming or disconfirming data for each of the propositions, and includes a brief summary of the evidence for each of the propositions. The narrative describing each of the propositions is presented below. In the initial propositions, success was defined in terms of sustainability, outcomes, and staff satisfaction. However, it became apparent that several ‘successful’ projects were not sustained for reasons that were outside the scope of the project. This is often termed ‘implementation failure’ [24] and needed to be separated from ‘project failure’. Moreover, as Martin *et al*. [33] point out, sustainability is part of a process or continuum, and not an end in itself.

As a result, we separated the concept of sustainability from ‘project success’ by defining project success in the following ways:

–implementation as planned

–goals achieved

–full local acceptance and adoption of the role

–codified practices that facilitate uptake of the role in a new site

–role/derivative of original model implemented in a new site

–local stakeholder understanding and support of the role

–appropriate use of role by stakeholders

–service benefits or efficiencies associated with new role

Several of the projects identified outcomes that improved as a result of the project, such as detailed patient health outcomes. While we attempted to verify the strength of these findings using standardized quality assessment tools [33], the heterogeneity of the data sources, quality of reporting, and methodologies used rendered this process ineffective. As this was a high-level process analysis we combined these outcomes together under the heading of patient outcomes and examined process issues that may be associated with this improvement. Consequently, this project cannot draw any conclusions about causality; it can only assess the nature of relationships between descriptive data.

Judgments about the propositions were made on the strength of the qualitative data arising from the projects. In some cases, the propositions lacked any supporting or refuting data, so could not be supported in this study.

After completing these steps the propositions were revised and the refined propositions are summarized in Table 4 outlined below. Note that we have substituted the term ‘success’ for ‘sustainability’ where appropriate. A single project illustration of the relationship between the project data and the propositions is presented in Additional file 2.

### Descriptive review of propositions

1.1 Better success of the new models of care is associated with early and full engagement of key stakeholders

Lack of engagement, hostility, and resistance by key stakeholders were important contextual barriers, while full engagement and commitment were key mechanisms for success. Five different types of resistant contexts were identified including:

–Staff unwillingness to change

–Lack of support from ‘powerful elites’. The most important single group identified here was the medical profession. However, lack of support from other important key stakeholders such as local managers, chief executives, project sponsors, and directors of allied health also prevented projects from proceeding or being successful.

–Interdisciplinary resistance arising from practitioners with equivalent status, but whose roles would be affected by the changes (for example, nurses were affected by changes to roles of AHPs and introduction of assistants).

–Resistance from other institutions

–Staff unwillingness to delegate.

Conversely, engagement of key stakeholders was an important driver of success. Of the completed projects the successful ones identified engagement with key stakeholders as a key factor for success; the eight unsuccessful projects all identified lack of engagement as a key cause. In one project involving two hospitals, one site was successful while the other failed; this was attributed entirely to the lack of support from medical stakeholders at the second hospital.

1.2 Better success of the new models of care is associated with bottom-up (rather that top-down) drivers

The importance of bottom-up drivers to the success of the new MoC was identified in several projects. ‘Bottom-up drivers’ refer to locally identified and owned reasons for introducing the new MoC, irrespective of whether this driver had a workforce, patient need, or service focus. This was substantiated in the following ways:

–Projects with evidence of success elsewhere were not able to be successful in contexts without a local champion to support the cause.

–Projects that did not clearly identify the local needs, drivers, or benefits, whether in terms of staff or patient needs, found it harder to gain traction.

Local engagement and ownership was particularly important for rural- projects. In situations where the project was not seen as a local priority and was driven by external expectations, projects had less success.

‘The project was not seen as a priority [by the local area]; allied health staff felt pressured by [name] to attempt unachievable deliverables.’ (Rural project)

1.3 Better success of the new models of care is associated with top-down support

Best *et al*. [34] found that having a blend of designated leadership and distributed leadership was likely to increase success of the target initiatives. They posit that engagement of individuals at all levels of the change process is required, and in particular, there needs to be an alignment between top leadership and distributed leadership. This suggests an alignment of the values, vision, and mission of the two types of leaders.

This proposition was supported at several levels of our study:

–A change of government towards the end of the MoC project resulted in a change of strategic priorities, uncertainty, and reallocation or cessation of funding. This change and uncertainty prevented the completion of some projects and prevented the sustainability of other successful projects due to realignment of priorities at policy and decision-making levels.

–Leadership in the form of change management training for staff, and support for AHA staff to undertake certificate training contributed to the success of projects.

–Strong executive sponsorship was associated with project success, whereas projects without executive support were discontinued or did not receive recurrent funding.

1.4 Better success of the new models of care is associated with legislative scaffolding

Support for the development and introduction of the new roles came from national and state initiatives. The recommendations in the *Australia*’*s Health Workforce Productivity Commission Research Report*[35] served as a driver and led to enterprise bargaining, which examined the scope of practice for AHA. However, in six cases the regulatory structure was not supportive of the required outcome. A disconnect was identified between the local initiative (such as extended scope of practice) and limits imposed by professional registration bodies.

1.5 Better sustainability of the new models of care is associated with codification of the processes, practices, and training used to implement the role

One of the major outputs from the QH MoC projects was the codification of processes necessary to implement new roles; specifically, this involved role definitions, service definitions, new competencies and competency frameworks, and new tools and pathways to support implementation of the new MoC. The codification of processes was important to help both the sustainability of the project in times of change, as well as the transfer of the project into a new environment/service/context. This was exemplified in one project, which specifically identified that having poorly defined role descriptions, objectives, and goals at the commencement of the project substantially slowed progress of the project; role descriptions were negotiated late in the project, delaying staff and patient recruitment.

Five projects used the Calderdale Framework [36] in the development of AHA roles. The Calderdale Framework facilitated service and task analysis to underpin role development, and the competency identification and training necessary to implement and sustain it. Projects using this framework were able to develop appropriate processes that were successfully implemented and sustained.

1.6 Better success of the models of care is associated with having powerful allies to drive the role forward

As previously noted, the engagement of key and powerful allies (predominantly medical champions) within an organization was paramount to the success of new roles and MoC. Support from these individuals can enable the necessary legislative changes required for acceptance of the new role and scope of practice by the regulatory body. They can also foster acceptance of the new MoC by others whose practice may be affected by the role changes.

1.7 Better success is associated with implementing new models of care that are appropriate for the context

The context into which the new MoC is implemented needs to be receptive to and supportive of the need for change (including local, geographic, population, clinical, professional, regulatory contexts). The characteristics of contexts that were identified as associated with successful implementation were:

–Willingness to discuss the potential to implement change. This required being flexible, open minded, and openly engaging with new ideas.

–Having a mindset that supports change.

–Willingness of team members to work with the change leader throughout the entire process.

–Ability to focus changes around those that will positively impact on patient outcomes.

–Having the relevant participants located in close proximity to each other.

A number of unsuccessful projects were initially started in contexts that were not receptive or supportive of the proposed changes and were discontinued; when moved to more supportive contexts that demonstrated the above characteristics, they were expected to be successful.

2.1 More efficient use of the role is associated with clearly defined roles within the model of care

There was evidence from 15 projects that having a clearly defined worker role within the MoC was associated with increased efficiency and sustainability of the role. In particular, it was important to have clearly defined role descriptions and identified training needs. In the absence of role clarity, protection of role boundaries was more likely to arise, as well as inefficient delegation of tasks to new practitioners. An important area requiring further clarity was the differentiation between advanced roles and full scope of practice.

2.2 More efficient use of the role is associated with clearly defined, understood, and unambiguous delegatory or allocatory models of care

The need for clearly defined delegatory or allocatory MoC was an important indicator of project success (8 projects). In particular, it was important to clearly identify tasks that could be delegated. Professional readiness for taking on extended practice varied between practitioners.

2.3 More efficient use of the role is associated with delegating practitioners having confidence in delegation and trust in the practitioner

Trust between practitioners was an important component of efficient delegation. This was supported by having a clear understanding of the roles, training, and competencies of the practitioners to whom they are delegating. Lack of trust resulted in a lack of confidence in the delegating practitioner, and consequently, in inefficient delegation.

2.4 More efficient use of the role is associated with allowing practitioners to work to their full scope of practice

Enabling staff to work to their full scope of practice, and providing structures to reinforce this was associated with increased efficiency (six projects). One of the strongest examples of this was the orthopedic podiatry triage service, which enabled podiatrists to work to their full scope of practice and led to substantial reductions in orthopedic surgery waiting lists and more efficient use of the orthopedic surgeon’s time.

Conversely, the inability or unwillingness of AHPs to delegate parts of their role to others, predominantly AHAs, resulted in inefficient use of the newly created roles. These challenges were related to:

–Lack of clarity with overlapping roles.

–Lack of understanding of how to delegate, and the need for education, supervision, and frameworks to support delegation. These structures needed to be in place from the beginning of the project.

–Lack of trust in assistants and engagement with the training provided to assistants by AHPs.

–Turf protection and reluctance to let go of some tasks to a workforce perceived to be less skilled.

The use of the Calderdale Framework assisted in resolving these challenges as it allowed AHPs to understand how they could improve their own patient care by delegating specific tasks and functions to AHAs.

3.1 Greater staff satisfaction is associated with models of care that provide for better career development opportunities

The implementation of new MoC improved career opportunities and led to greater staff satisfaction. Surveys to measure staff satisfaction with the new MoC were undertaken in some projects as part of their evaluation processes. The following outcomes were identified:

–Development of more sustainable roles within the organization.

–Perceived ability by allied health team members to complete more quality activities, therapy interventions and patient education, thus improving their job satisfaction.

–More appropriate task to skill matching.

–Increased sense of achievement through the learning of new skills and acquiring a broader knowledge base, particularly for AHA.

–A perception of inclusion into a multidisciplinary team.

–Improved staff morale.

However, in projects where turbulent leadership, poor recruitment to leadership roles, and an influx of junior/inexperienced staff were reported, the staff satisfaction with the new MoC was poor. These barriers overshadowed the positive impacts the new MoC may have had on team members.

3.2 Greater staff satisfaction is associated with role clarity

The data did not demonstrate a direct link between increased staff satisfaction and role clarity. However, there was evidence that improved understanding and acceptance of the new roles arose, in part, through appropriate consultative processes that were reinforced through processes of role clarity. This is consistent with findings from other studies that examined role clarity issues associated with role boundaries for workplaces with multi-disciplinary teams [37]. Issues of role clarity were also upheld through union negotiations.

Better understanding and acceptance of new roles was associated with: 1) AHPs having the increased knowledge and ability to identify appropriate tasks for the proposed assistant role, 2) building trust in delegation models and a multi-professional assistant workforce, and 3) changes that resulted in more advanced AHP and advanced AHA positions being incorporated into services to improve efficiencies and reduce costs. Staff acceptance of these new roles within the teams was demonstrated in six projects with the majority of these projects being continued.

This proposition is, therefore not upheld in this context.

3.3 Greater staff satisfaction is associated with seeing value in/impact of the new models of care

Appreciating the value and impact of a new role is largely associated with the new MoC being driven by a locally identified need or being driven from the bottom-up. The other main factor impacting on this is engagement. Both of these factors are described under Proposition 1.

3.4 Greater staff satisfaction is associated with appropriate support for the development and implementation of the new models of care

This factor is also closely related to the importance of engagement of key stakeholders from across a multitude of levels within the organization, and therefore, is closely related to Proposition 1.

4.1 Better patient outcomes are associated with greater engagement of patients in the decision making associated with their care delivery

Few of the projects reported the patient perspective; therefore, there is limited data to assess this proposition. Failure to facilitate patient engagement was highlighted in one unsuccessful project; the patients using the service did not feel that there was a need for additional services and therefore did not use them.

4.2 Better patient outcomes are associated with putting the patient at the center of the new models of care, rather than the practitioner

This study found evidence that if patients were not involved in driving the new services then patient outcomes were less likely to be the specific focus of the project, or were not improved. In particular, one project, driven from the top-down, with the goal of reducing unplanned hospitalizations in a pediatric population was not successful because the patients (parents) did not see a need for more services, and therefore did not make use of the new services. Another project used a multidisciplinary team to shift the approach from professionally centred care to patient centred care. Some projects were focused on decreasing wait times for patient and improving patients safety; however, the outcome evaluations were generally based on the staff’s perceptions that patient outcomes improved.

4.3 Better patient outcomes are associated with providing *any* care or service where the alternative is no service, or a long waiting list

The data from this study predominantly related to providers and identified outcomes from the provider perspective; however, there was some empirical evidence to support this proposition. Eleven projects provided care or services where previously, there was limited or no service provided due to long waiting lists or geographic inequities in service distribution. However, because of the lack of data collected to describe outcomes from the patient’s perspective, we recommend that in future workforce change projects, data are captured that focus on the patient’s perspective of new MoC.

## Discussion

This project identified and tested empirically a series of propositions associated with successful workforce change. The propositions that were supported by the data are listed below:

1. Better sustainability of a new MoC is associated with:

•full engagement of all key stakeholders first

•bottom- up drivers (rather than top-down)

•top-down support to drive, underpin, and sustain the new MoC

•legislative scaffolding to reinforce the new MoC, including award and pay structures supported in industrial agreements, and ratified at the highest possible levels of government, to avoid undermining by professional boundary arguments

•codification of the processes, practices, and training used to implement the role

•powerful allies to drive the role forward

•implementing new MoC that are appropriate for the context (local, geographic, population, clinical, professional, and regulatory)

2. More efficient use of health practitioner roles is associated with:

•clearly defined roles within the MoC

•clearly defined, understood, and unambiguous delegatory or allocatory MoC

•delegating practitioners having confidence in their delegation, which comes from understanding the roles, training, and competencies of the practitioners to whom they are delegating

•trust, derived from time and exposure to the new MoC , is important for establishing appropriate delegation/collaboration/referring practices

•allowing practitioners to work to their full scope of practice and having structures that reinforce this.

3. Greater staff satisfaction is associated with:

•better career development opportunities

•appreciating value / impact of the role

•appropriate support for the development and implementation of the MoC.

4. Better patient outcomes are associated with:

•greater engagement of patients in the decision making associated with their care delivery

•putting the patient at the centre of the MoC, rather than the practitioner

•providing any care or service where the alternative is no service, or a long waiting list

These propositions have been further synthesized into three broad principles of workforce change:

(1) Drivers for change need to be closely linked to clinical practice and patient care. Workforce change needs to be driven by perceived or potential benefits to patients, staff and /or services at the local level.

(2) The context for workforce change must be supportive at all levels. This includes a supportive legislative and industrial environment, professional environment, and leadership and champions.

(3) Mechanisms for workforce change should include engagement of key stakeholders, access to resources to support the implementation and performance of the role, a facilitated change management process, and appropriate governance and support structures.

The first two propositions had the greatest volume of data to support them. While there was evidence to support the propositions within statements 3 and 4, they were not mutually exclusive to these domains, and tended to reinforce the statements within Propositions 1 and 2.

The strength of the evidence largely reflects the nature and volume of data collected relating to each factor. In other words, ‘weak’ evidence was likely to be due to a lack of available data rather than findings that refuted the propositions. We were surprised by (a) the lack of patient engagement and (b) the lack of patient focus in the projects. Consistent with the literature in this field, the majority of the projects focus on professions and changing role boundaries or developing new MoC. This focus, by definition, leads to interdisciplinary challenges and rivalry because the emphasis of change becomes the renegotiation of roles, rather than the best way of allocating care to meet the needs of the patient. The lack of a clear patient focus meant that goals were often process-based, rather than outcome-focused.

This project developed and tested a novel method, Inductive Logic Reasoning, to create and empirically test theories of workforce change. This method has the advantage that it enabled us to develop and develop theories from the existing literature to create a series of propositions that were then tested empirically and transparently against a large dataset. This method aimed to address some of the limitations of the existing theory-based evaluation approaches by using logic models in a transparent way to develop theories of change which can be transparently tested. The dataset incorporated a large range and volume of data sources of varying structure, content, and quality, and we were able to transparently extract data against pre-defined themes to create the initial logic model.

This study involved the analysis and synthesis of a large volume of data from a range of sources, in varying formats, and over a short period of time. The combination of the logic model and proposition development and testing appears to have been an effective and transparent way to arrive at a high-level synthesis of the data, which was the goal of this study. However, in achieving that goal, we have lost a great deal of depth and detail of the raw data.

One of the challenges of the evaluation was identifying measures of success due to the heterogeneity of the projects and their large range of potential impacts. We were unable to draw firm conclusions about the outcomes of the workforce projects for reasons identified above; however, there were clear process indicators associated with project success. The nature of the evidence of change makes it difficult to draw causal relationships.

As with all research, there is the risk of researcher bias. Workforce research is highly contextually dependent, and we have framed this research within the Australian health workforce context. This approach automatically biased the researchers towards certain norms. We attempted to ensure objectivity by embedding our propositions within the literature first, and then testing them empirically against the data arising from the projects; however, the research still has the Australian health-care context as its normative setting.

The project reports used as data were not written with the expectation that they would form part of a large-scale evaluation; hence, their findings were presented in different ways. We have triangulated a range of primary and secondary data sources from a variety of participants to ensure the validity of our findings.

We acknowledge that methods to empirically develop and test change theories require further development and refinement. For instance, it may be possible to draw tighter conclusions if we were to re-assess each project against the success criteria. This would also help to validate the success criteria.

## Conclusion

The implementation of new MoC is a complex process and broad principles of change management apply. Based on the findings of this study, we developed a comprehensive Workforce Change Checklist: An Evidence Based Practice Guide for Implementing Successful Workforce Change [38], which is available from the authors on request. This tool draws together the data developed in the logic model, propositions and principles in a tool to support workforce change agents, funders, and commissioners to deliver successful workforce change projects.

## Abbreviations

AHAs: Allied health assistants; AHPs: Allied health professionals; CASP: Critical Appraisal Skills Programme; EPOCH: The Cochrane Collaboration’s Effective Practice and Organisation of Care; ILR: Inductive logic reasoning; MoC: Models of care; QH MoC: Queensland Health Models of Care.

## Competing interests

Kerry Vanniekerk-Lyons was employed as a project officer by Queensland Health and was involved in the project management of the overall Models of Care projects. Her role in this research has been to help broker access to the data, provide support for the multiple site specific research governance requirements, and provide clarification of facts relating to the data. To maintain objectivity, the remaining members of the research team, who were external to the Queensland Health MoC processes, undertook the majority of interpretation and analysis of the data and have no competing interests.

## Authors’ contributions

SN conceived the original project, obtained the funding, developed the methodology and was involved in the data analysis and synthesis and drafted the paper. AR was overall project manager and was responsible for much of the data management, synthesis and analysis. AM and SG were involved in the data analysis and synthesis. KV provided ethics and governance support. All authors provided input into the conceptual development and ongoing drafts of the paper. All authors read and approved the final manuscript.

## Authors’ information

Susan Nancarrow, Director of Research at the School of Health and Human Sciences, Southern Cross University, Australia.

Alison Roots and Anna Moran are research fellows at the School of Health and Human Sciences, Southern Cross University, Australia.

Sandra Grace is a senior lecturer at the School of Health and Human Sciences, Southern Cross University, Australia.

Kerry Vanniekerk-Lyons is a full-time PhD student examining sustainable workforce change at the School of Health and Human Sciences, Southern Cross University, Australia.

## Supplementary Material

Additional file 1 Data extraction template for Queensland Health documents. Click here for file

Additional file 2 **Single case study of the introduction of a social work assistant illustrating data output from the Google form**[39]. Click here for file

Additional file 3 Queensland Health models of care project summaries. Click here for file

Additional file 4 Summary of project outcomes. Click here for file

Additional file 5 Proposition summaries and supporting evidence. Click here for file
