# Supplementary figures and images for: Implementing large-scale workforce change: learning from 55 pilot sites of allied health workforce redesign in Queensland, Australia
Source: Hum Resour Health. 2013 Dec 11;11:66. doi: 10.1186/1478-4491-11-66 (PMC3895764; doi:10.1186/1478-4491-11-66)

### Additional file 1: Data Extraction Template for QH docments

###
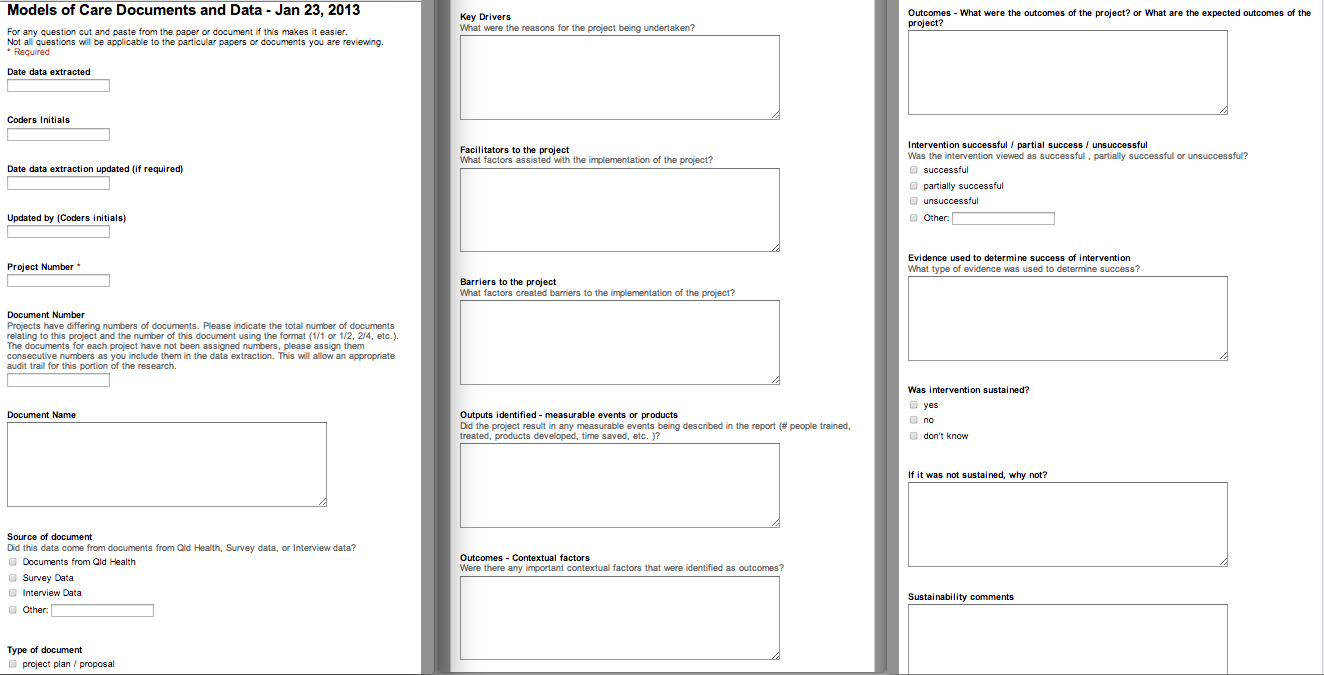

Supplement: Additional file 1 — Data extraction template for Queensland Health documents. [file 1478-4491-11-66-S1.docx]
