# Supplementary material for: Implementing large-scale workforce change: learning from 55 pilot sites of allied health workforce redesign in Queensland, Australia
Source: Hum Resour Health. 2013 Dec 11;11:66. doi: 10.1186/1478-4491-11-66 (PMC3895764; doi:10.1186/1478-4491-11-66)
Supplement: Additional file 2 — Single case study of the introduction of a social work assistant illustrating data output from the Google form[39]. [file 1478-4491-11-66-S2.docx]

### Additional file 2: Single case study of the introduction of a social work assistant illustrating data output from the Google form.

| **Timestamp** | 1/24/2013 15:00:37 |
| --- | --- |
| **Date data extracted** | 1/24/2013 |
| **Coders Initials** | AR |
| **Date data extraction updated (if required)** |  |
| **Updated by (Coders initials)** |  |
| **Project Number** | 14 |
| **Document Name** | Social work assistant |
| **Document Number** |  |
| **Source of document** | Documents from Qld Health |
| **Type of document** | Final report from a project |
| **Was this a phase 1, phase 2, or dual phase, project?** | Phase 1 (2009- 2011) |
| **Category addressed by the project (primary)** | Allied health assistant support role |
| **Category (secondary)** | Service or role redesign |
| **Setting for the project** | Hospital |
| **Setting - population** | Urban centre |
| **Type of service involved - direct patient services** | Acute care in-patient ward, long term care / aged care facility, home care services |
| **Type of service involved - technical services** |  |
| **Type of health care professional involved** | Social worker |
| **Type of role of practitioner** | Delegated |
| **Project details** | Project aimed to increase social work (SW) productivity, optimise social work service through more efficient alignment of skill to task, improve staff satisfaction and enhance the quality of the patient journey. The project phase involved development and validation of a contextualised and discipline specific Social Work Assistant (SWA) position description using Lean®-based mapping processes. The implementation phase saw the establishment of a new SWA role within the hospital SW team. The sustainability phase included the effective roll-out of implementation workshops to SW teams across Queensland, and other successful strategies employed locally, state-wide and nationally. All of the SW leaders attending those workshops indicated intent to implement an SWA role within 2 years.  In this context, SWs are key professionals who facilitate timely and appropriate patient discharge from adult medical beds into residential aged care facilities and into the community with necessary services. They also provide high level assessment, counselling and crisis intervention for patients and families.  This SWA role operates within a delegation model which enables SWs to operate at full scope with increased job satisfaction. This model describes SW support tasks within a competency framework, and meets safety and quality concerns with clinical governance, delegation, clarity of role and clinical supervision. The new model allows SWs more time to carry out higher complexity tasks, whilst ensuring patient safety, quality and consistency of service.  Although the project did not plan for this it resulted in a role re-design for SW's at the hospital. |
| **Geographical scope of project** | One location in Qld |
| **Patient Focus** | Adult patients to residential aged care facilities and into the community with necessary services. |
| **What type of change did the project involve?** | Top down change, Radical change |
| **Key Drivers** | In 2009 the Social Work Director identified a number of issues with Social Work service delivery in the acute adult setting. These included: a large amount of staff time was spent on low level tasks; social workers were not working to full scope of practice; there was inconsistency in approach to processes and tasks; staff were dissatisfied with the level of work they were able to perform. |
| **Facilitators to the project** | - The health service has a very positive staff culture, within an industrially and professionally stable atmosphere. - A cohesive funding structure with reasonable autonomy in the hands of the service director, enabling flexibility, with the possibility of embedding workforce redesign changes. - The Executive Sponsor was responsible in envisioning the project, and in strongly and actively guiding project activity. - The Executive Sponsor was also supported by experienced, stable and effective team leaders, two of whom sat on the SWA Steering Committee. - Good quality and clear communication and processes were possible. - The absence of any Welfare Office or similar roles allowed for both a Social Work workforce hungry for the opportunity for redesign to enable their scope of practice, as well as producing a less complicated context for the delineation of roles. - The appointment of an experienced Project Manager with knowledge of Allied Health Assistant role development. |
| **Barriers to the project** | - An interesting barrier to the delegation process was identified as the „egalitarian‟ ethos of Social Work professionals within the team, who with no previous experience in delegating to a support role were initially reluctant to delegate the lower level tasks which had been clearly identified as within the scope of the SWA role. - Expansion and sustainability of project limited by barriers around funding or budgetary flexibility for the creation of the new SWA positions. |
| **Outputs identified - measurable events or products** | - 20% increase in number of new patients seen over the period of the trial, even after adjustment for additional FTE. - Cost per occasion of service decreased by 11%. - 50% of SW team now have formal change management training - Time in Lieu Accrual for SW team members dropped by an average of 47% - 100% of staff 'strongly agreed' that SWA made a positive difference in optimising SW service and productivity; 100% staff agreed or strongly agreed made a positive difference in enhancing patient journey and staff satisfaction. |
| **Outcomes - What were the outcomes of the project? or What are the expected outcomes of the project?** | Increased staff satisfaction; increased scope of SW practice; increased productivity; decreased costs; sustainable model; robust methodology; discipline wide potential; recurrent funding |
| **Intervention successful / partial success / unsuccessful** | Successful |
| **Evidence used to determine success of intervention** | Key Performance Indicators - standardised measures relating to client health, service productivity, human resources developed by MOC AHA projects.  Once a viable model for the new role had been delivered, applicability across other sites in Qld Health was undertaken.  Directors of Social Work at twelve (12) sites formally completed expressions of interest in workshops to create knowledge and skills in design of Social Work Assistant roles. |
| **Was intervention sustained?** | Yes |
| **If it was not sustained, why not?** |  |
| **Sustainability comments** | For sustainability, the Social Work profession, within QLD Health, and nationally, should be engaged as key stakeholders in a wider change management strategy. Sustainability strategies were employed across three “spheres”: within The Health Services, within QLD Health, and nationally.  The primary sustainability efforts were focussed on ensuring that the single SWA role established by the project at the Adult Hospital within the Social Work team was sustainable, by “getting it right‟, that is, by;   - establishing “best practice” role design processes - recruiting carefully - assiduously refining the SWA role within the team in ways that ensured it was effective - building skills and awareness in redesign and the benefits possible through training, communicating and engaging MAH staff to grow a sense of ownership of the SWA role collecting data that irrefutably demonstrated the value of the role to the team, and their patients - committing resources to sustainably fund the SWA.   It was also important to ensure that the SWA role would be accepted and replicated in QLD by ensuring key influencers and decision-makers were informed and satisfied that the SWA role was an effective, safe, and SW profession-friendly innovation through: ·   - alignment with existing and developing assistant workforce models and initiatives - establishment of a model that allayed fears through excellent clinical governance - a program of clear and detailed engagement with the SW Directors - successful cooperation with the Allied Health Assistant State-Wide Steering Committee around an approved SWA Role description, the AHA Audit process and the likely inclusion of an SWA role in the industrial framework - cooperation with AHPOQ in the filming of a high quality DVD showcasing the model broadcasting the details and success of the model through conference presentations and state-wide video-links - engaging AHPOQ and securing funding for a series of SWA implementation workshops across the state - securing resources to sustainably fund the SWA position, in partnership with QLD Health.   It was also considered important to ensure that the SWA role could be accepted and replicated across the SW workforce by:   - increasing the likelihood of creation of an SWA qualification through engagement of the training body - engaging key SW leaders nationally by direct briefing of the association and key SW academics. |
| **Propositions** |  |
| **Any issues that relate to the sustainability of the model of care identified in this project?** | Full engagement of stakeholders, top-down support, legislative "scaffolding", codification of the processes, practices, training, appropriate for the context |
| **Any issues that relate to the efficiency of the model of care identified in this project?** | Clearly defined roles, clearly defined and understood delegatory / allocatory model, delegation practitioners have confidence in delegation, trust with the delegation/ collaboration / referring patterns, practitioners allowed to work to full sop |
| **Any issues that relate to the staff satisfaction with the model of care identified in this project?** | Role clarity, value and impact of the role is recognized, support for the development and implementation of role is present |
| **Any issues that relate to patient outcomes associated with the model of care identified in this project?** | Providing any care or service when the alternative is no service or a long waiting list, increased access to care |
| **Are areas for further research identified?** | The Social Work Task Complexity Hierarchy is an innovation and deserves further validative and developmental research.  Advanced practice role for social workers is recommended. |
| **Are there any exceptions or observations that are of particular relevance to our models?** | The strongest limitation to an AHA performing tasks across many professions within the one team was the need for good clinical supervision, and balancing the demands of each profession within the team.  The experience within this project is that perhaps up to 8 professional staff would provide the critical mass of tasks to keep a full-time SWA occupied, though this needs validation in other sites and contexts.  SWA roles can only operate in a delegation model, which is consistent with other AHA roles across the Allied Health workforce and there is no evidence that AHA roles which currently exist in other professions “take away” professional duties.  One issue deserving of specific mention, which looms as a barrier to further implementation of this reform, is the expressed sense by social work leaders that program funding models which currently exist may have created a barrier to flexibility of workforce redesign and a barrier to change. Social work leaders within QLD Health may need to develop a consistent vision and clear strategies and mechanisms to negotiate what is required in terms of regaining a greater degree of self-determination with their funding models.  At a national level, the profession has committed to scoping the creation of an SWA qualification, and the SWA project will be key informants of that process. The president of the association expressed strong support for the role and invited the project team to brief the board in 2011. A key SW academic expressed interest in collaborating in further development and validation of the Social Work Task Complexity Hierarchy.  The SWA project has therefore in 18 months sustainably inserted an entirely novel role into the SW workforce at a site, jurisdictional and national level, and taken every step possible to ensure the role’s longevity in that workforce. |
| **Other comments** | The findings of this project have been published [[39](#_ENREF_39)]. |
