# Supplementary material for: Implementing large-scale workforce change: learning from 55 pilot sites of allied health workforce redesign in Queensland, Australia
Source: Hum Resour Health. 2013 Dec 11;11:66. doi: 10.1186/1478-4491-11-66 (PMC3895764; doi:10.1186/1478-4491-11-66)
Supplement: Additional file 3 — Queensland Health models of care project summaries. [file 1478-4491-11-66-S3.docx]

### Additional file 3: Queensland Health Models of Care Project Summaries

| **Project ID** | **Project Name (de-identified)** | **Location (Rural / regional / metro)** | **Project Aim** | **Practitioner Type**  **(if specific)** |
| --- | --- | --- | --- | --- |
|  | ***Advanced practice role for existing staff (AHP)*** |  |  |  |
| **1** | Rehabilitation MoC project - Advanced AHP in Rehab | Metropolitan | Develop advanced AHP role | PT |
| **2** | Allied Health Clinical Lead - (Phase 1 project re-designed) | Metropolitan | Evaluate the effects of an allied health clinical lead on patient journey, flow and service integration. | OT |
| **3** | Advanced AHP trial Emergency (ER) | Metropolitan | Develop an Advanced AHP role in ER | SW, OT, PT |
| **4** | Advanced AHP trial Acute Aged Care | Metropolitan | Develop an Advanced AH role on Medical Ward | SW, OT, PT |
| **5** | Acute Medical Services Model of Care | Regional | Develop Advanced AHP role using learning from Phase 1 project | OT, SW |
| **6** | Musculoskeletal pathway prescribing | Metropolitan | Prescribing and admin of anti-inflammatory medications | PT |
| **7** | Prescribing Trial - Botox for Neurology by PT / OT | Metropolitan | Develop role for prescribing / administering Botox | OT / PT |
| **8** | *Advanced AHP - Aged care* | Regional | Developed advanced AHP role supported by AH assistant role | OT/ PT/ Psychologist |
|  | ***Full scope of practice*** |  |  |  |
| **9** | Telerehabilitation ( eHAB) | Regional | Integrate the use of eHAb services, delivery of services to rural clients, improve access in cost effective manner, effectiveness of techno in providing clinical support and supervision | OT, PT, Speech Pathology - rural based practitioners |
| **10** | Designing & implementing an innovative MoC in Mental Health | Regional | Develop a new service delivery model for existing clinicians; work to full scope of practice | Mental health clinicians |
| **11** | Radiography Abnormality Description | Metropolitan | Improve radiographer ability for abnormality description, improve timely reporting; | Radiographers |
|  | ***New Support Role*** |  |  |  |
| **12** | AHA in internal medicine - Eat- Walk- Engage Program | Metropolitan | Trial of new role | AHA |
| **13** | Pharmacy Assistant (PA) in Renal Unit | Metropolitan | Validate use of PA to maximise patient medication understanding/ minimise medication waste | Pharmacy Assistants (PA) |
| **14** | SW Assistant Trial Project | Metropolitan | Introduce assistant role to improve patient flow and decrease job dissatisfaction | Social Work Assistant |
| **15** | Redesigning ear nose and throat care paths through Innovative audiology practice | Metropolitan | Develop assistant role | Audiology Assistant |
| **16** | Developing partnerships to improve patient journey from isolated sites | Rural / remote | Build partnerships to streamline patient journey for people with chronic diseases from isolated sites |  |
| **17** | AHA Project | Metropolitan | 6 different trials of 12 different assistant roles across diverse settings – hospital / community |  |
| **18** | Use of AHA for service provision in rural areas | Rural / remote | Train a AHA across group of AHP to improve service provision | General AHA |
| **19** | Advanced AHA - rural outreach model | Rural / remote | Develop a role to support rural / remote practitioners | AHA - Chronic Disease |
|  | ***Other - Educational (AHP)*** |  |  |  |
| **20** | AHP / student partnership - in chronic disease management | Regional | Improve access for patients | Students - Dietetics, Exercise Physiology, OT, Podiatry, Pharmacy, SW |
| **21** | Student assisted clinic in Child Development Clinic | Regional | Feasibility of student clinic model for child development clients | Psychology |
| **22** | Clinical Measurement Practitioner | Regional | Develop a graduate program for sleep sciences, respiratory, cardiac and neurology services ; Define graduate competencies | Clinical Measurement Practitioner |
|  | ***Service - Role Redesign*** |  |  |  |
| **23, 24** | High Intensity Aphasia Clinics | Metropolitan | Trial three methods of increasing intensity of aphasia services | Speech Pathology |
| **25** | Advanced AHP in persistent pain management | Metropolitan | Re-design of clinic services | PT, OT, Psych |
| **26** | Teleradiography | Metropolitan / Rural | Trial of supervision of licensed operators using telehealth | Radiography |
| **27** | Allied Health Supportive Care Initiative (Palliative care) | Metropolitan | Improve AH support for Advanced Care planning | PT, OT, SW, Pharmacy, Dietitian, AHA |
| **28** | Paediatric Outpatient service delivery | Metropolitan | Improve wait time, simply referral process, enhance info flow, reduce duplication | Multi-disciplinary team |
| **29, 30** | Allied Health Coordinated Respiratory Outpatient Support Service (ACROSS) | Metropolitan | Improve health outcomes for children with chronic conditions | Nutrition/Dietetics, OT, PT, Pharmacy, SW |
| **31** | Hospital to Home Outreach for Malnourished Elderly | Metropolitan | Identify barriers and enablers to new MoC. Refocus dietitians time for increased discharge planning and post hospital follow up by partnering with, training and supporting community service providers, | Dietitian - community agencies /support workers |
| **32** | AH Assistant | Metropolitan | Developed roles with new or higher level tasks | Nutrition/Dietetics, OT, PT, Speech Pathology |
| **33** | Rural Remote outreach - Central West - Advanced AHA in rural health | Rural outreach | Explore development of advanced role using telehealth | AHA |
| **34** | AHA | Rural / remote | Support existing professional staff, provide more consistent & cost-effective services to all communities | Multi-disciplinary AHA |
| **35, 36** | Medical Imaging Assistant (MIA) | Metropolitan | Develop career pathway, tasks and education for MIA | Medical Imaging |
|  | ***Service -Role Re-design/ Advanced level role for existing support staff (AHA)*** |  |  |  |
| **37** | New Models of pharmacy practice, Pharmacy Assistants roles | Rural / remote | Expand scope of PA to be in line with overseas countries | Pharmacy tech / assistants |
| **38** | New Models of pharmacy practice, Pharmacy Assistants roles | Metropolitan | Expand scope of PA to be in line with overseas countries | Pharmacy tech / assistants |
| **39** | Advanced AHA in management of diabetes & renal disease | Regional | Develop and trial an appropriate and sustainable MoC for managing aspects of chronic disease including development of an AHA role | Podiatry, Dietetics |
| **40** | Occupational Therapy service re-design - medical wards / Community | Regional | Service re-design to provide in-reach to hospital from community - OT assistant in hospital / OT in community | OT/ OT Assistant |
|  | ***Transprofessional/ Integrated - skill development (AHP)*** |  |  |  |
| **41, 42, 45, 46** | Rural Generalist AHP | Rural / remote | Create a rural generalist AHP role - own discipline then pick up skills from other disciplines, e.g. OT /PT; Transprofessional practice / shared competencies | PT, OT, SW, Pharmacy, Dietitian |
| **43** | Allied Health (AH) service to ER through mobile AH Team in-reach | Rural / remote | Used Calderdale Framework to develop shared competencies and delegated practice | Allied health team |
| **44** | New MoC for Community Rehab services | Rural / remote | Transprofessional care support for self-management models for chronic disease | AHP |
|  | ***Triage / Treatment (AHP)*** |  |  |  |
| **47** | General Paediatrics AH Screening Service | Metropolitan | Screening and intervention service | AHP |
| **48** | Orthoptist led Ophthalmic Clinic | Metropolitan | Screening and intervention service | Orthoptist |
| **49** | Oral Health Service for Adults with diabetes mellitus | Metropolitan | Improve oral health care to patient with diabetes mellitus | Oral Health Therapist, Dentists |
| **50** | Paediatric Podiatry | Regional | Study to determine need for statewide paediatric podiatry clinic | Podiatry |
| **51** | Virtual Foot Ulcer ER Services | Metropolitan | Feasibility of podiatry services in ER | Podiatry |
| **52, 53, 54** | Orthopaedic Podiatry Triage Clinic (OPodTC) | Metropolitan | Manage non-urgent foot problems with conservative interventions; Phase 2 - statewide implementation | Podiatry |
| **55** | Persistent Pain Advanced AHP - Pain Clinic | Regional | New clinic - triaging / assessing - taking on larger role with advanced Scope of practice | PT, OT, Psych |
| **Did not participate** | Neurological screening for spinal pain | Metropolitan | Improve timely access, decrease waitlists, develop a non-surgical pathway, improve patient and provider satisfaction | PT, pharmacy, dietitian, OT, psychologist |
| **Did not participate** | Pathology, Forensic & Scientific services | Statewide | Training of 3 new roles - Analytical assistant, Cross-trained specimen collection / reception officer, operational staff supervisor | Laboratory staff |
| **Did not participate** | Exploring rights to private practice - Paediatric Therapy services | Regional |  |  |
| **Did not participate** | Dietetic led Gastroenterology clinic | Metropolitan | Triage of patients for Gastro-Enterology consultant | Dietitian |
