# Supplementary material for: Implementing large-scale workforce change: learning from 55 pilot sites of allied health workforce redesign in Queensland, Australia
Source: Hum Resour Health. 2013 Dec 11;11:66. doi: 10.1186/1478-4491-11-66 (PMC3895764; doi:10.1186/1478-4491-11-66)
Supplement: Additional file 4 — Summary of project outcomes. [file 1478-4491-11-66-S4.docx]

### Additional file 4: Summary of Project Outcomes

|  | **YES** | **NO** | **Comment** |
| --- | --- | --- | --- |
| Projects that were successful | 11(1 site), 12, 13, 14, 16, 17, 19, 20, 23/24, 26, 27, 28, 32, 37, 38, 39,48, 49, 51, 52-54 | 3,4,7, 9, 10,11(1 site), 29, 35, 36 | - 2 projects from phase 1 failed but moved into phase 2 as re-designed projects; - Phase 2 projects still underway; - Technology challenges, lack of staffing; - Ahp resistance to expanded aha role (turf protection); - Project did not have a identified patient need |
| Projects that were sustained | 11, 12, 13, 14, 16, 17, 23/24, 20, 27, 28, 32, 48, 52- 54 | 19, 26, 34, 37, 38, 39, 49, 51 | - Sustainability dependent on demonstration of efficiencies & cost savings, new funding source needed for continuation; - Issues with staff retention, generic role too broad, need to focus on only 1-2 disciplines; - Some projects never designed to be sustained; - More training required for advanced assistant roles; - Supervision and training issues; - Appeared very successful but discontinued due to lack of executive support; - Scoping for project only, no funding |
| Projects in progress | 1,2,5,6,8, 15, 18,25, 40, 41, 43, 44, 46, 47,51, 55 |  |  |
| Projects that were replicated | 12, 13, 14, 23/24, 48, 52 – 54 |  | - Expanding to other wards; - Planning to expand across state; - Trying to be replicated at 12 sites; - Planned expansion |
