# Supplementary material for: Implementing large-scale workforce change: learning from 55 pilot sites of allied health workforce redesign in Queensland, Australia
Source: Hum Resour Health. 2013 Dec 11;11:66. doi: 10.1186/1478-4491-11-66 (PMC3895764; doi:10.1186/1478-4491-11-66)
Supplement: Additional file 5 — Proposition summaries and supporting evidence. [file 1478-4491-11-66-S5.doc]

Additional file 5: Proposition Summaries and Supporting Evidence

**Proposition 1: Better Sustainability of Model of Care is associated with:**

| **Factors influencing sustainability** | **Projects with positive evidence supporting proposition** | **Refuting data** | **Evidence / Comments** |
| --- | --- | --- | --- |
| 1.1 Full engagement of stakeholders | 1, 2, 5, 11, 12, 13, 14,16, 17, 19, 20, 27, 28, 32, 37, 38, 48, 52, 53, 54 |  | *Calderdale Framework:* Use facilitated sustainability and was found helpful  *Stakeholders:* Stakeholder analysis undertaken – to identify potential facilitators/barriers and work done to enhance /alleviate; training, involvement & integration of key stakeholders very important (e.g. medical, nursing, AHS, health information, educators); success requires significant involvement, engagement and understanding from large number of stakeholders; stakeholders need to understand the difference between advanced scope and expanded scope; need for clear communication with all stakeholders re barriers; too many competing priorities prevent key stakeholders from investing necessary time in new MoC;  Expanded scope roles are more difficult to enact;  AHP need to support the full use of AHAs;  Newer AHPs more receptive, enthusiastic and creative;  New MoC need to be supported by clinical staff and have an identified ‘patient problem’ that they are to address (if staff /patients don’t think there is a need, the new MoC won’t succeed); |
| 1.2 Bottom-up drivers | 1, 2, 5, 10, 17, 23/24, 31, 39, 49, 52, 53, 54 |  | Projects more successful if driven from quality of care and workplace pressures issues;  Bottom-up drivers need top-down support;  Bottom-up driver created ownership of new MoC;  Most projects had top down drivers to define Scope of practice, reduce costs, increased quality of patient outcomes;  Role needs to be designed with input from local AHPs;  Most successful changes in services/roles were bottom-up driven. |
| 1.3 Top-down support | 2, 5, 13, 14, 19, 20, 23/24, 25, 27, 47, 48 |  | Supported at state and federal level with funding;  Support for new MoC required from top levels of hospital/ heath district; Executive sponsor strongly active in guiding projects (lack of executive support – new MoC discontinued);  Early focus on identification of potential resources to continue new MoC;  Funding not available to continue new MoC |
| 1.4 Legislative “scaffolding” | 1, 5, 14, 51 |  | Current regulation can prohibit new MoC/ Recognised need for regulatory changes;  Legislative changes need to be undertaken first;  Negotiations needed with unions and regulatory bodies;  Need for national standards for roles; |
| 1.5 Codification of processes, practices, training | 1,5, 6, 11, 12, 14,15, 18, 20, 32, 37/38, 52, 53, 54, 55 |  | New practices should be codified through development of role descriptions, short courses with formalised credentials developed, and policy framework to support all these processes;  Clearly defined scope of practice ensured robust measurements of success of new MoC;  Need for scope of practice, role statements/ boundaries to be defined and endorsed by all;  Governance model developed;  Training packages developed;  Training AHA roles can be time consuming for AHPs, do not expect to be done on site by AHPs;  Need for consistent standards, titles, remuneration for AHA roles;  Need to define differences between basic scope, full scope and advanced scope AHA roles;  Processes fully documented. |
| 1.6 Powerful allies | 11, 19, 20, 27, 32, 48, 52, 53, 54, 55 |  | Success or failure of project can be related to degree of medical / nursing support;  Support from medical champions;  AHPs resistant to development of career progression for AHA roles when they do not have their own career progression. |
| 1.7 Appropriate for context | 1,5, 14, 15, 16, 19, 20, 26, 48 |  | Environment too complex to enact extended scope of practice role when other supports are not present;  Generalist roles in rural areas more difficult to maintain due to recruitment, training, supervision concerns;  New MoC need to meet individual community needs;  AHA roles easier to establish in urban areas;  Site/local ownership of new role important for success of MoC;  Equipment, training, and support needs to be appropriate for context;  Fear of change;  Organisation needs to be ready for change;  Don’t try to take on too much change at one time;  Used to assist future recruitment to rural areas. |
| Other | 6 |  | Liability concerns factor in project failure  Need for many agreements when change involves community based individual practice owners  Supervision issues – framework for supervision helpful |

**Proposition 2: More / less efficient use of the role is associated with:**

| **Factors influencing efficiency** | **Projects with positive evidence supporting proposition** | **Refuting data** | **Evidence / Comment** |
| --- | --- | --- | --- |
| 2.1 Clearly defined roles | 1, 5, 12, 13, 14, 15, 16, 17, 25, 32, 37, 38, 39, 46, 52, 53, 54, 55 |  | Role description and training needs identified, including training manuals need to clearly defines roles;  Use of Calderdale Framework improved role definitions and identified trans-disciplinary tasks;  Administrative & clinical support roles need to be defined separately;  Negotiated partnerships improve role clarity;  Role / turf protection can occur;  Defining the meaning of full scope versus advance scope important - how scope of practice is defined can limit use / expansion of the role;  Improved role definitions can lead to role reclassification and improved remuneration;  Lack of professional experience impeded clear role definitions;  Time spent understanding current roles leads to clearer descriptions of new roles (limited time prevents roles from being appropriately defined);  Teamwork required to scope, purpose and positioning of interdisciplinary AHP roles;  Role defined early in development of new MoC;  Stepping outside of own discipline can be challenging. |
| 2.2 Clearly defined and understood MoC (Bottom-up drivers) | 1, 5, 12, 14, 16, 17, 23/24 32, 47, 48, 52, 53, 54 |  | Tasks for delegation identified;  Professional readiness for taking on extended roles varies between practitioners;  Role underutilized due to resistance to new AHA role;  AHA role to utilise more technology so less supervision is required;  Roles lacked clear understanding of scope of practice;  Lack of experience impacted on success with new MoC;  Top down support for new MoC enhanced bottom-up drivers;  Transprofessional MoC still in trial – limited results available. |
| 2.3 Delegating practitioners have confidence in delegation | 5, 14, 23/24, 48, 52, 53, 54 | 7, 8, 11 | Lack of confidence in delegation of clinical tasks;  Need to have credentialed practitioner to support delegation of clinical duties;  Instigation of formal governance arrangements help with delegation ;  Supervision and delegatory frameworks improve confidence in delegation;  Transprofessional MoC still in trial – limited results available. |
| 2.4 Trust based on time and exposure to new MoC | 5, 14, 23/24, 48, 52, 53, 54 | 3/4, 7, 32 | Despite time in the role trust with some AHA roles did not improve;  Lack of support-> lack of trust;  Transprofessional MoC still in trial – limited results available. |
| 2.5 Practitioners allowed to work to full scope of practice | 13, 14, 31, 44, 46, 48, 52, 53, 54 | 3/4, 34 | Support staff encouraged to work to full scope and therefore minimise duplication of services;  Executive level support to work to full SoP;  Increasing demand for services supporting working to full scope;  Support from medical champions;  AHP did not have confidence in roles that cross professional boundaries;  Allowed time of other practitioners to be re-prioritised  Transprofessional MoC still in trial – limited results available. |
| Other |  | 11 | Concerns re risk of delegation to uncertified practitioner |

Proposition 3: Greater staff satisfaction is associated with

| **Factors influencing staff satisfaction** | **Projects with positive evidence supporting proposition** | **Refuting data** | **Comments / Evidence** |
| --- | --- | --- | --- |
| 3.1 Better career development opportunities | 2, 5, 16, 25, 32, 35, 36, 44, 46, 48, 50, 52, 53, 54, 55 |  | Advancement opportunities enhanced (leadership roles);  Advanced AHA were “keener” on roles than AHP, believed they lead to increased career opportunities;  New leadership positions created;  Rural generalist role enhanced sustainability of AHP services in community (development of HP5 level role);  Potential for improvement in recruitment and retention |
| 3.2 Role clarity | 32, 52, 53, 54 |  | Multiple recruitments / large amount of sick leave indicative of high level of stress |
| 3.3 Seeing value / impact of role | 8, 9, 11, 14, 15, 16, 17, 19, 23/24, 27, 32, 37, 38, 46, 52, 53, 54 |  | Medical staff see value in role;  Surveys indicated staff and stakeholders found value in new role;  New access to technology appeared to provide staff satisfaction;  Surveys indicated staff valued new services;  Enhanced collaboration between staff in rural /remote areas;  Staff recognising the importance of working together and intervening earlier with patients;  Development of skills that are transferable across sites in rural areas |
| Appropriate support for development/ implementation of role | 1,2,5, 9, 13, 14, 15, 16, 18, 19, 23/24, 27, 32, 39, 44, 46, 48, 50, 52, 53, 54, 55 |  | Active support provided for role developments;  Associated training programs enhanced staff’s ability to provide patient centred care;  Staff surveys indicate high level of support for new role;  Surveys indicated staff found satisfaction with new services;  Improved staff integration |

**Proposition 4: Better outcomes are associated with:**

| **Factors influencing patient outcomes** | **Projects with positive evidence supporting proposition** | **Projects with negative evidence supporting proposition** | **Comments / Evidence** |
| --- | --- | --- | --- |
| 4.1 Greater patient engagement in decision making | 13, 15 |  | Few of the reports had a patient focus or reported a patient perspective |
| 4.2 Patient-centred MoC | 10, 13, 15, 16, 18, 28, 29/30, 35, 36, 39, 44 |  | Decreased wait times for patients improved patient safety;  MoC developed based on consumer need, however no follow-up study done to confirm effectiveness;  Teamwork created a shift from professional centred approach to patient centred |
| 4.3 Providing any care or service where the alternative is no service | 1,8, 9, 14, 16, 17, 18, 19, 23/24, 25, 27, 31, 32, 46, 47, 48, 49, 52, 53, 54 |  | Creation of AHA role increased access to care;  Increasing demand has created a situation where little to no service is available to many patients |
| Other | 11, 13 |  | Project identified gaps in the current systems that could provide potential risks for health care system;  Improved patient safety, addition of AHA role allowed increased direct patient care to be provided by AHP. . |
